# Cryptococcosis and tuberculosis co-infection in mainland China

**DOI:** 10.1038/emi.2016.95

**Published:** 2016-09-07

**Authors:** Min Chen, Abdullah MS Al-Hatmi, Yuchong Chen, Yang Ying, Wenjie Fang, Jianping Xu, Ferry Hagen, Nan Hong, Teun Boekhout, Wanqing Liao, Weihua Pan

**Affiliations:** 1 Department of Dermatology, Shanghai Key Laboratory of Molecular Medical Mycology, Shanghai Institute of Medical Mycology, Changzheng Hospital, Second Military Medical University, Shanghai 200003, China; 2 Directorate General of Health Services, Ministry of Health, Ibri Hospital, Ibri 21-115, Oman; 3 Department of Medical Mycology, CBS-KNAW Fungal Biodiversity Centre, Utrecht 3584CT, The Netherlands; 4 Department of Dermatosurgery, Shanghai Dermatology Hospital, Shanghai 200050, China; 5 Beijing Institute of Radiation Medicine, Beijing 102206, China; 6 Department of Biotechnology, McMaster University, Hamilton, ON L8S 4L8, Canada; 7 Department of Medical Microbiology and Infectious Diseases, Canisius-Wilhelmina Hospital, Nijmegen 6532SZ, The Netherlands

Dear Editor,

Cryptococcosis is a fungal infection caused by members of the *Cryptococcus neoformans*/*C. gattii* species complex,^1^ which causes approximately one million infections among HIV-infected patients per year, with approximately 625 000 annual mortalities among HIV/AIDS-associated deaths worldwide.^2^ Tuberculosis (TB), which is currently the second leading cause of death from infectious diseases in HIV/AIDS patients, is one of the deadliest communicable diseases and is caused by *Mycobacterium tuberculosis*. In China, accurate epidemiological data on cryptococcosis is not available at present, but the number of reported cryptococcosis cases has increased over the last two decades.^3^ Approximately 10% of global TB cases occurred in mainland China in 2014.^4^

Both cryptococcosis and TB are life-threatening infections with a global distribution, are strongly associated with HIV infection and share similar clinical manifestations.^5^ Little is known regarding the features of cryptococcosis/TB co-infection, particularly in mainland China, where cryptococcosis, TB and HIV/AIDS are increasingly important. Dozens of cryptococcosis/TB co-infection cases have been reported in mainland China, mostly since 2000. The majority of those cases were published in Chinese journals, and thus, the cases are not easily accessible to non-Chinese readers. Here, we describe a retrospective analysis of cryptococcosis/TB co-infection cases with a confirmed diagnosis in mainland China.

We systematically searched the medical records from our hospital and literature databases, such as PubMed and China National Knowledge Infrastructure. The criteria for the diagnosis of cryptococcosis included a positive cryptococcal culture, positive India ink staining, and/or a positive cryptococcal antigen titer in a clinical specimen. A diagnosis of TB was established in cases with a positive *M. tuberculosis* culture from clinical specimens. A cryptococcosis/TB-co-infected patient is defined as a TB patient who was diagnosed with cryptococcosis within the two months before or 6 months after the diagnosis of TB.^5^

A total of eight cases (one from our hospital and seven from the literature databases) were collected and analyzed (Table 1 and Supplementary Figure S1). All cases were reported since 2002, and they were distributed in the following six cities: Nanchang (*n*=3); Beijing (*n*=2); Nanning (*n*=1); Chongqing (*n*=1); and Shanghai (*n*=1). The mean age was 35.75 (ranging from 4 to 62), and males were predominantly infected (M/F=7/1). One patient was confirmed as HIV-positive, and another was diagnosed as having type 2 diabetes mellitus. Regarding the profiles of the co-infections, the most frequent was tubercular meningitis combined with cryptococcal meningitis (CM) (4/8), followed by tubercular meningitis plus pulmonary tuberculosis combined with CM (2/8). None of them were documented as having been exposed to pigeon droppings; one patient had close contact with acute TB patients 3 months before the onset of TB.

Regarding the clinical manifestations, fever (8/8), headache (5/8) and meningeal irritation (5/8) were the most frequent symptoms and signs. India ink staining (6/8) and positive cultures (7/8) were the main approaches used to diagnose cryptococcosis, and the cryptococcal antigen (CrAg) test was only performed in the case from our hospital. All cases of TB were confirmed by positive culture. On the basis of the timing of the diagnosis of cryptococcosis and TB, the co-infections can be divided into two types: (i) concurrent co-infection (cases 1, 6 and 8), indicating that the two infections were diagnosed simultaneously; and (ii) sequential co-infection (TB first: cases 2, 3, 4 and 5; cryptococcosis first: case 7), which were diagnosed as cryptococcosis occurring within the 2 months before or 2 months after the diagnosis of TB. Among the sequentially co-infected patients (*n*=5), most (4/5) were diagnosed with TB first, and cryptococcosis was diagnosed at an average of 41.75 days later (ranging from 7 to 70). Only one patient was first diagnosed with cryptococcosis, and TB was diagnosed 49 days later. Taken together, our analyses suggest that seven of the co-infection cases had delayed diagnoses (*n*=4) or erroneous diagnoses (*n*=3). The therapeutic protocols used to treat the co-infections were not described in detail in most of the reported cases. Regarding TB treatment, multiple drugs were combined for the anti-TB treatments in all of the co-infection cases, including isoniazid (INH), rifampicin (RIF) and pyrazinamide (PZA). The adopted cryptococcosis therapies were also variable.

Recently, co-infections such as HIV/TB have been considered as a new battleground in the struggle to control HIV/AIDS worldwide.^6^ In our opinion, cryptococcosis/TB co-infection also should be taken seriously because cryptococcosis and TB are both closely linked to HIV/AIDS, and the co-infection has likely been severely underestimated. To our knowledge, the first case of a cryptococcosis/TB co-infection was reported in the United States in 1951.^7^ Over the past 20 years, such co-infections have been more frequently reported worldwide, including co-infections in both HIV-positive and HIV-negative patients.^5^ In contrast, there have been few reports of cryptococcosis/TB co-infections in the Chinese population in English journals. A recent single-center case series from Taiwan reported 23 TB/cryptococcosis co-infections, raising significant concerns over its burden in mainland China.^5^ Regarding the epidemiological characteristics of cryptococcosis/TB co-infection in mainland China, the majority of these cases were reported in cities with relatively advanced medical facilities. Remarkably, some regions in mainland China, such as Henan and Xinjiang provinces, have a tremendous TB burden.^8^ We hypothesize that the lack of reports from these regions was likely due to their limited medical resources, causing delayed or incomplete diagnosis. Compared with some of the major cities, the medical resources in these regions are very limited. Thus, patients with the co-infection might have a delayed or incomplete diagnosis. Similar to a previous study,^5^ males were more frequently reported as having TB and cryptococcosis co-infections in the current study. This result is not surprising because previous surveys have shown that males are the predominant patients who are infected with either cryptococcosis or TB.^5^ At present, the detailed molecular epidemiology of strains causing cryptococcosis/TB co-infections in China are not known. The etiological agents of cryptococcosis in cryptococcosis/TB patients were identified at the species level in studies from Canada (*C. deuterogattii* AFLP6/VGII) and Guatemala (*C. liquefaciens*).^9, 10, 11^ Regarding the co-infection profiles of the organ involved, the most frequent combination was TB (brain) with CM (brain), followed by TB (brain and lungs) combined with CM. Thus, the brain was the organ that was most frequently involved in the co-infections examined in our survey, followed by the lungs.

Among the eight cases, seven were reported to have a delayed or incomplete diagnosis. Recently, a lateral flow assay for cryptococcal antigen (CrAg) detection has been recommended by the WHO as the preferred approach for diagnosing cryptococcosis.^12^ However, in most of the cases included (7/8), this technique was not used. Until now, no clinical guidelines for the treatment of cryptococcosis/TB co-infected patients have been proposed.^13^ Regarding the anti-TB treatments among co-infected patients, drug susceptibility testing should be performed before treatment begins because of the increasing prevalence of multidrug-resistant TB.^4^

In conclusion, our study on cryptococcosis/TB co-infections in mainland China should raise awareness and improve our understanding of this co-infection. The lack of specific symptoms has likely resulted in a severe underestimation of co-infections because many of these co-infected patients could have a missed or delayed diagnosis. Physicians should consider the possibility of cryptococcosis/TB co-infection as a masked infection, particularly in HIV-infected patients who have received regular, but unsuccessful, anti-TB or antifungal treatments. A multicenter, prospective, and multidisciplinary clinical survey of cryptococcosis/TB co-infections should be conducted in parts of the world with serious TB, cryptococcosis and HIV/AIDS burdens, notably many sub-Saharan African and Asian countries.

## Figures and Tables

**Table 1:** Epidemiological and clinical characteristics of eight cases of cryptococcosis/TB co-infection in mainland China

**Case**	**Chinese DOI**	**Date**	**Sex**	**Age**	**Underlying diseases**	**Pigeon contact**	**TB contact**	**Cities**	**Affected site of the co-infection**	**Time interval from TB to crypto**	**Delayed or erroneous diagnosis**	**Anti-TB treatment**	**Antifungal treatment**	**Prognosis**
1	10.3760/cma.j.issn.1672-7088.2003.15.040	02 Aug	F	4	No	No	No	Chongqing	Crypto (blood)+TB (liver)	Concurrent	Erroneous	INH+ RIF+SM+ PZA	AmB+FCZ	Survived
2	10.3760/j.issn:0578-1426.2005.09.025	03 Jan	M	16	No	No	Yes	Beijing	Crypto (brain)+TB (brain)	7 Days	Delayed	INH+ RIF+ PZA	AmB+FCZ+intrathecal injection of AmB	Survived
3	10.3969/j.issn.1009-8194.2005.10.034	05 Jul	M	48	No	No	No	Nanchang	Crypto (brain)+TB (brain+lung)	40 Days	Delayed	INH+EMB+RIF+PZA	Intrathecal injection of AmB+FCZ +5-FC	Survived
4	10.3969/j.issn.1009-8194.2005.10.034	05 Aug	M	62	No	No	No	Nanchang	Crypto (brain)+TB (brain+lung)	70 Days	Delayed	INH+EMB+RIF+PZA	Intrathecal injection of AmB	Deceased
5	10.3969/j.issn.1009-8194.2005.10.034	05 Oct	M	30	No	No	No	Nanchang	Crypto (brain)+TB (brain)	50 Days	Delayed	INH+EMB+RIF+PZA	Intrathecal injection of AmB+FCZ	Survived
6	10.3760/j:issn:0376-2491.2007.27.017	05 Oct	M	56	Diabetes mellitus type 2	No	No	Beijing	Crypto (blood+skin)+TB (blood)	Concurrent	Erroneous	Died before anti-TB treatment	FCZ	Deceased
7	10.3969/j.issn.1671-6450.2009.09.007	09 Apr	M	39	HIV-positive	No	No	Nanning	Crypto (brain)+TB (brain)	−49 Days	No	INH+ SM+ RIF+ PZA	AmB+5-FC	Survived
8	Case from our hospital	05 Mar	M	31	No	No	No	Shanghai	Crypto (brain)+TB (brain)	Concurrent	Erroneous	INH+RIF+PZA+EMB	AmB+5-FC	Survived

Abbreviations: 5-fluorocytosine, 5-FC; amphotericin B, AmB; cryptococcosis, Crypto; ethambutol, EMB; fluconazole, FCZ; isoniazid, INH; no dat, ND; para-aminosalicylic acid, PAS; pyrazinamide, PZA; streptomycin, SM; rifampicin, RIF; tuberculosis, TB.

Note: Chinese DOI (http://www.chinadoi.cn/portal/index.htm).
