# Supplementary figures and images for: Cryptococcosis and tuberculosis co-infection in mainland China
Source: Emerg Microbes Infect. 2016 Sep 7;5(9):e98–. doi: 10.1038/emi.2016.95 (PMC5113057; doi:10.1038/emi.2016.95)

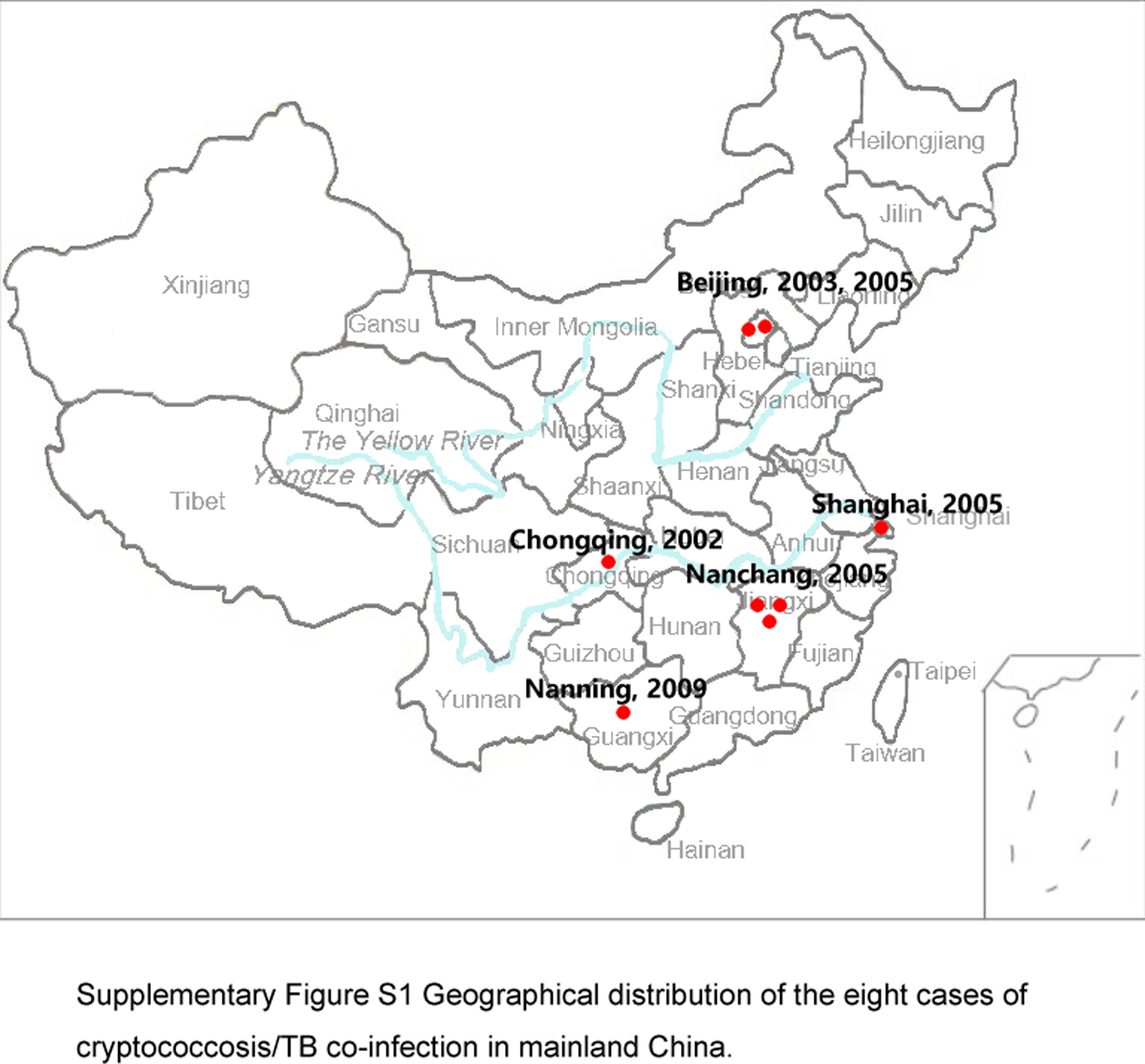

Supplement: Supplementary Figure 1 [file emi201695x1.tif]
